# Is it safe to withhold long-term anticoagulation therapy in patients with small pulmonary emboli diagnosed by SPECT scintigraphy?

**DOI:** 10.1186/s12959-016-0086-0

**Published:** 2016-05-31

**Authors:** R. Ghazvinian, A. Gottsäter, J. Elf

**Affiliations:** Lund University, Division of Vascular Medicine, Skåne University Hospital, Ruth Lundskogs Gata 10, S-205 02 Malmö, Sweden

**Keywords:** Withholding conventional AC therapy, V/P SPECT, Small PE, Subsegmental pulmonary embolism

## Abstract

**Background:**

The need for anticoagulation therapy (AC) in patients with subsegmental pulmonary embolism (SSPE) diagnosed by computed tomography of the pulmonary arteries (CTPA) has been questioned, as these patients run low risk for recurrent venous thromboembolism (VTE) during 3 months of follow-up. Whether this applies also to patients with small PE diagnosed with pulmonary scintigraphy has not yet been evaluated, however.

**Methods:**

We therefore retrospectively evaluated 54 patients (mean age 62 ± 19 years, 36 [67 %] women) with small PE diagnosed by ventilation/perfusion singe photon emission computed tomography (V/P SPECT) who did not receive conventional long-term AC.

**Results:**

More than half of our patients (36[67 %]) received less than 48 h of AC, 11 (20 %) patients were treated for 2–14 days, and 7 (13 %) for 15–30 days. The majority (28 [52 %]) of our patients had a non-low simplified pulmonary emboli severity index (S-PESI), and 7 (13 %) had malignancy. D-dimer was negative in 18 (33 %), positive in 10 (19 %), and not analyzed in 28 (52 %) patients. Phlebography of the lower extremities had been performed with negative result in one patient.

During 90 days of follow up no deaths or PE occurred. Seven patients were readmitted to hospital, whereof two (2/54 [4 %]) were diagnosed with deep venous thrombosis (DVT) necessitating AC therapy.

**Conclusion:**

In conclusion, withholding longterm AC therapy in patients with SSPE diagnosed by V/P SPECT resulted in 4 % risk for recurrence of VTE during 90 days of follow up, and can therefore currently not be recommended.

## Background

The annual incidence rates of deep venous thrombosis (DVT) and pulmonary embolism (PE) are approximately 0.5–1 per 1000 inhabitants [1]. The clinical presentation of PE extends from asymptomatic patients to shock states, but most patients (95 %) are normotensive and present with breathing difficulties or pleuritic pain [2]. The use of multiple-detector computed tomography pulmonary angiography (CTPA), has led to an increased number of diagnosed sub-segmental pulmonary emboli (SSPE), accounting for around 5–15 % of PE cases [3].

Albeit CTPA is considered the primary diagnostic tool for detection of PE and is available 24 h a day, 7 days a week, there are alternative methods [4]. Using planar ventilation-perfusion lung scan (V/Q scan), the majority of patients with suspected PE have non-diagnostic examinations [5]. Prospective management studies demonstrated that patients with low or intermediate probability V/Q scan, low pretest probability of PE, and negative compression ultrasonography can be safely managed without anticoagulation [6, 7]. Lung scintigraphy has been further developed, however. Modern ventilation/perfusion single photon emission computed tomography (V/P SPECT) and “holistic” interpretation criteria reduce the risk for non-diagnostic findings, and increases the sensitivity and specificity for diagnosis of PE [8]. With this technique PE can be classified as segmental or sub-segmental and graded according to the percentage of the pulmonary vascular bed affected [8].

Some diagnostic challenges persist, however. Even though the use of multi-detector CTPA or V/P SPECT in combination with diagnostic algorithms as for example the Wells score [9] has increased the diagnostic accuracy of PE, the mortality of the disease has remained consistent [10–12]. This might indicate that many of the small SSPE treated after detection with CTPA may be of low clinical significance [4]. In fact, treatment of SSPE in patients with high bleeding risk, and low risk for recurrent venous thromboembolism (VTE) might perhaps even be contraindicated due to increased risk of bleeding and other treatment complications. Whether it is safe to withhold anticoagulant (AC) treatment in patients with SSPE diagnosed by CTPA and negative bilateral ultrasound of the legs is currently evaluated in an ongoing trial [13], but whether this approach is applicable to patients with small PE diagnosed by V/P SPECT has not yet been assessed.

In 898 patients diagnosed with PE by V/P SPECT, we previously reported risk of recurrence and bleeding in 307 patients with small PE undergoing home treatment with AC [14]. We now report a retrospective follow-up of patients with PE diagnosed by V/P SPECT in whom conventional long-term AC therapy for different reasons had been withheld.

## Methods

All consecutive 898 patients with acute PE diagnosed by V/P SPECT between 2007 and 2011 at Lund University Hospital, Sweden were included in a prospective registry [14]. V SPECT had been performed after inhalation of aerosolized 99 m TC-Technegas to the lung in the supine position with acquisition lasting for 11 min, and P SPECT with a dual-head gamma camera in the supine position after i.v. administration of 50 MBq 99mTc-macroaggregated albumin. V SPECT illuminates ventilated areas and leaves areas with reduced/absent ventilation on the imaging screen, whereas P SPECT illuminates areas where blood flow is normal but leaves areas without perfusion on the imaging screen. PE was diagnosed and quantified by counting segments showing complete or relative mismatch between ventilation and perfusion defects [8].

After diagnosis, patients were clinically assessed according to a pre specified clinical algorithm [14]. Conventional long-term AC treatment defined as therapeutic doses of low molecular heparin or vitamin-K antagonist for at least three months according to current guidelines [1, 12] was withheld by the clinician if the V/P SPECT result was interpreted as falsely positive for technical reasons (non diagnostic for PE), if the perfusion defect was thought to represent an old and no longer clinically relevant embolization, or if the embolization was thought to be too clinically irrelevant to merit treatment. Withholding of long-term treatment also required that patients were hemodynamically stable, did not have clinical signs or symptoms of DVT, and that the extent of perfusion defects in the V/P SPECT images was less than ≤ 20 % of the pulmonary vascular bed. Based on these criteria, 54 patients (6 %, mean age 62 ± 19 years, 36 [67 %] women) did not receive conventional long-term AC therapy. AC treatment of shorter duration was, however, given to many of these patients during the diagnostic work-up as summarized in Table 1. Baseline data, clinical characteristics including retrospective calculation of sPESI score [15], additional imaging procedures, quantification of perfusion defects on V/P SPECT, d-dimer, and troponin T/brain natriuretic peptide (NT-BNP) results were collected from patient files. Readmissions, recurrent VTE, and death during 90 days after the final dose of AC therapy were assessed by review of hospital records and imaging databases.

**Table 1:** Baseline characteristics and duration of anticoagulant (AC) therapy in 54 patients with small pulmonary embolism diagnosed by ventilation/perfusion single photon emission computed tomography (V/P SPECT) who did not receive conventional long-term AC. N(%)

Predisposing factors for thrombosis n(%)	
Malignancy	7 (13)
Oral contraception	1 (2)
Surgery or immobilization	5 (9) (one with malignancy)
Trauma	1 (2)
Previous venous thromboembolism	4 (7)
Pregnancy	2 (4)
Concomitant diseases n(%)	
Congestive heart failure	18 (33)
Chronic obstructive pulmonary disease or asthma	9 (17)
Investigations n(%)	
D-dimer	28 (52), negative in total 18 (33)
NT-proBNP	9 (17), negative in total 8 (15)
Troponin T	28 (52), negative in total 24 (44)
Venous ultrasound or phlebography	1 (2), negative in total 1 (2)
CTPA	17 (31), positive in 6 (11)
Risk stratification n(%)	
sPESI score 0	12 (22)
sPESI score 1	28 (52)
sPESI score 2	11 (20)
sPESI score 3	2 (4)
sPESI score 4	1 (2)
Duration of anticoagulant therapy n(%)	
<48 hours	34 (63)
2–14 days	11 (20)
15–30 days	7 (13)
31–90 days	2 (4)

## Results

### Background data

Twenty (37 %) patients had a predisposing factor for VTE; active cancer, previous surgery, immobilization, pregnancy, contraceptive pill use, trauma or previous DVT (Table 1). Data on concomitant diseases, sPESI scores, and the number of auxiliary investigations such as D-dimer, compression ultrasound, contrast phlebography and CTPA are also shown in Table 1.

### Follow-up

**Fig. 1:**
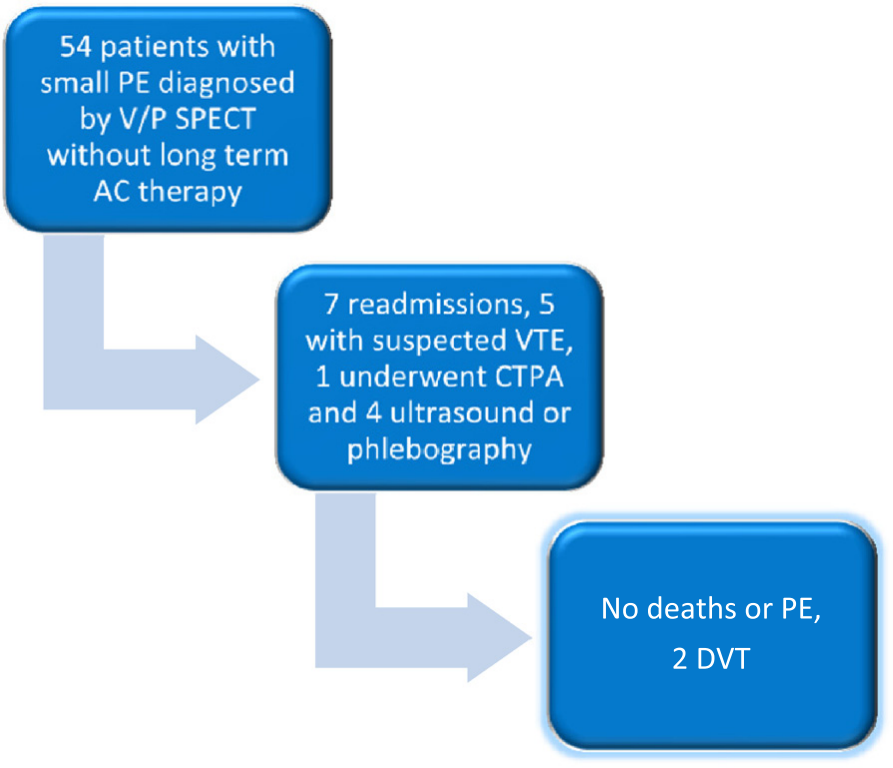
Flow diagram of study. PE = Pulmonary embolism, V/P SPECT = Ventilation/Perfusion single photon emission computed tomography, AC = Anticoagulant, VTE = Venous thromboembolism, CTPA = Computed tomography pulmonary angiography, DVT = Deep vein thrombosis

No deaths occurred during 90 days of follow up after the final dose of AC therapy. Seven patients (13 %) were readmitted to hospital, in 5 cases (9 %) for suspected VTE. One patient (2 %) underwent CTPA, without signs of PE. Four patients (7 %) underwent phlebography or ultrasound of the lower extremities, whereof two (2[4 %]) were diagnosed with DVT necessitating long-term AC therapy (Fig. 1).

One 71 year old man who had received AC for 24 h was readmitted 38 days after the final AC dose because of swelling of the left leg, and ultrasound showed DVT extending up to the external iliac vein provoked by plaster cast immobilization due to a tibial fracture. One 92 years old woman who had received AC for 20 days was readmitted 52 days after the final dose of AC therapy due to swelling of the right leg. Ultrasound showed DVT extending up to the common femoral vein.

## Discussion

To our knowledge, this is the first retrospective study in which withholding of conventional long-term AC therapy has been clinically evaluated in patients with a diagnosis of small PE made by V/P SPECT. We report two cases of DVT during a follow up period of three months (4 %), but no PE or death. These figures are comparable higher than what has been reported when AC therapy has been withheld in SSPE patients diagnosed by CTPA. Goy et al retrospectively study reported no recurrences (0 %) among 30 patients but that treatment provoked major bleeding in 2/43 patients treated with AC [3]. Moreover, Carrier and co-workers summarized no recurrences in 60 patients with SSPE diagnosed with CTPA in combination with a negative compression ultrasonography [4], when reviewing data from four different reports [10, 16–18]. These low numbers must be compared with the results of den Exter et al who reported that 4/116 (3.5 %) patients with SSPE treated with AC had a recurrent VTE during 3 months follow up challenging the assumption that SSPE patients have a low risk profile and better clinical outcome compared to patients with more proximal PE [19].

In a cross-sectional survey on clinician’s opinions on SSPE [20], it was shown that physicians are comfortable with withholding of therapy if the 3 month risk for recurrent VTE is <2 %. This means that our algorithm with a cutoff of <20 % extension of perfusion defects in the V/P SPECT images leads to a rate of VTE recurrence that would not be considered acceptable for the majority of clinicians. Whether the use of a lower limit of PE extension on V/P SPECT such as for example <5 % or <10 % would have resulted in a lower risk for recurrent DVT remains to be systematically investigated. The extension of the perfusion defects on V/P SPECT in our two patients later diagnosed with VTE was <10 %, and use of this cut-off would therefore not have resulted in any VTE diagnoses during follow-up in our material.

Whether a normal result on bilateral lower extremity ultrasonography should be requested before AC therapy is withheld in patients with confirmed small PE on V/P SPECT also needs to be further evaluated. Whereas this strategy has been used after SSPE diagnosis with CTPA [3] and is currently under further scientific evaluation [13], none of our two patients later diagnosed with DVT had undergone ultrasound or phlebography when the decision to abstain from long-term AC therapy was made.

Whether results of the s-PESI score [17] can be used to predict which patient with small PE that will develop VTE during follow-up is still unclear. Retrospectively calculated s-PESI scores differed in the two patients with VTE during follow-up in our study; zero in the male patient and three in the female patient. Our male patient had an elevated D-dimer which was thought to be caused by concomitant disease, whereas D-dimer was not assessed in the female patient. The combination of Wells score [9] and D-dimer in the diagnostic work-up of PE [1, 12] is only applicable in outpatients without too many comorbidities.

The results of our study underlines the importance of the ongoing prospective study [13] performed to evaluate the safety of withholding AC therapy in patients with SSPE. It is important to note, however, that the results of this trial will only be applicable in patients in whom PE is diagnosed by CTPA and in whom serial ultrasound of the lower extremities is negative. Our results indicate that the future study results cannot be extrapolated to patients with small PE diagnosed by V/P SPECT, at least not without routinely performing bilateral compression ultrasonography of the lower extremities.

The discrepancies in results between our and previous [3, 4, 10, 16–19] might also be caused by differences in the composition of the study populations. When relating our results to previously published data [10, 15–17, 21], our patients were of comparable age (62 years vs 65 and 56 years in the studies of Goy et al. [3] and den Exter et al [19]). The prevalence of malignancy was lower in our study (13 % vs 28 and 18 %) respectively, however. Furthermore, one third of our patients had congestive heart failure (CHF) and 17 % had chronic obstructive pulmonary disease (COPD), figures higher than the 9 % for both CHF and COPD reported by den Exter et al. [19].

Among patients with malignancies and PE, the risk for recurrent VTE is increased among those with symptomatic PE, compared to those with incidentally detected asymptomatic disease [20]. In our study, however, no VTE was detected during follow-up of the 7 patients with known malignancies.

The duration of AC treatment given during diagnostic work-up and initial hospital care is another important issue. In the majority (63 %) of our patients who received less than 48 h of AC therapy, one case of VTE occurred, whereas the other case of VTE was diagnosed in the group (13 %) of patients receiving 15-30 days of AC therapy. The risk of recurrence during the first three months is approximately 2 % [22], and the concept of generally prescribing one month of AC to patients diagnosed with small PE with V/P SPECT, thereby limiting the risk of bleeding, also needs to be further investigated.

The aim of our retrospective study was not to compare V/P SPECT with CTPA in diagnosis of small PE, but PE diagnosis made on V/P SPECT nevertheless had been reassessed with CTPA in 31 % of patients, and was confirmed in a third of these. This reflects that the clinical decision to withhold long term AC therapy was made in three different situations after a positive V/P SPECT; when the result was believed to be non diagnostic, when the perfusion defect was thought to represent a no longer clinically relevant embolization, and when the embolization was thought to be clinically irrelevant. The reason to perform CTPA is most evident in the first of these three scenarios, and in 11 of our patients the PE diagnosis was dropped after a negative CTPA. In the 6 patients in whom long-term AC therapy was withheld in spite of positive results on both V/P SPECT and CTPA, therapy was withheld by the clinician for the third of the above reasons.

A recent paper comparing diagnostic performance between CTPA and V/P SPECT reported that these modalities were equivalent with respect to receiver operating curves (ROC) analysis, with areas under curve (AUC) of 0.99 (95 % CI 0.96–1.00) and 0.98 (95 % CI 0.94–1.00) for V/P SPECT and CTPA, respectively [23].

The main study limitation is of course that we conducted a retrospective clinical follow-up, and not a randomized prospective study. Furthermore, as the decision to withhold conventional long-term AC therapy was made on different ground, our patient material is heterogeneous. Nevertheless, we think that it represents practice in an often occurring difficult clinical situation.

## Conclusions

Withholding of conventional long term AC therapy in patients diagnosed with small PE with V/P SPECT was associated with a 4 % risk of VTE diagnosis during 3 months of follow-up. This would not be considered acceptable for the majority of clinicians, and the concept can at the present stage therefore not be recommended.

## Abbreviations

AC, anticoagulation; AUC, area under curve; CHF, chronic heart failure; COPD, chronic obstructive pulmonary disease; CTPA, computed tomography pulmonary arteries; DVT, deep vein thrombosis; PE, pulmonary embolism; ROC, reciever operating curves; S-PESI, simplified pulmonary emboli severity index; SSPE, subsegmental pulmonary embolism; V/P SPECT, ventilation/perfusion single photon emission computed tomography; V/Q scan, ventilation-perfusion lungscintigraphy; VTE, venous thromboembolism.
